# The association of cognitive coping style with patient preferences in a patient-led follow-up study among colorectal cancer survivors

**DOI:** 10.1007/s00520-024-08758-y

**Published:** 2024-08-01

**Authors:** Kelly R. Voigt, Lissa Wullaert, M. H. Elise van Driel, Max Goudberg, Pascal G. Doornebosch, Jennifer M. J. Schreinemakers, Maria Verseveld, Koen C. M. J. Peeters, Cornelis Verhoef, Olga Husson, Dirk J. Grünhagen

**Affiliations:** 1 Department of Surgical Oncology and Gastrointestinal Surgery, Erasmus MC Cancer Institute, Doctor Molewaterplein 40, 3015 GD Rotterdam, Netherlands; 2 Department of Medical Oncology, Netherlands Cancer Institute, 1066 CX Amsterdam, The Netherlands; 3 Department of Surgery, IJsselland Hospital, Capelle aan den IJssel,, The Netherlands; 4 Department of Surgery, Amphia Hospital, Breda, The Netherlands; 5 Department of Surgery, Franciscus Gasthuis, Rotterdam, The Netherlands; 6 Department of Surgery, Leiden Universitair Medisch Centrum, Leiden, The Netherlands

**Keywords:** Follow-up, Colorectal cancer, Personalised care, Coping style

## Abstract

**Introduction:**

Amidst the rising number of cancer survivors and personnel shortages, optimisation of follow-up strategies is imperative, especially since intensive follow-up does not lead to survival benefits. Understanding patient preferences and identifying the associated patient profiles is crucial. Coping style may be a key determinant in achieving this. Our study aims to evaluate preferences, identify coping styles and their associated factors, and explore the association between coping style and patients’ preferences in colorectal cancer (CRC) follow-up.

**Methods:**

In a prospective multicentre implementation study, patients completed the Threatening Medical Situations Inventory (TMSI) to determine their coping style. Simultaneously patients choose their follow-up preferences for the CRC trajectory regarding frequency of tumour marker determination, location of blood sampling, and manner of contact.

**Results:**

A total of 188 patients completed the TMSI questionnaire after inclusion. A more intensive follow-up was preferred by 71.5% of patients. Of all patients, 52.0% had a coping style classified as ‘blunting’ and 34.0% as ‘monitoring’. Variables such as a younger age, female gender, higher educational level, and lower ASA scores were associated with having higher monitoring scores. However, there were no significant associations between follow-up preferences and patients’ coping styles.

**Conclusion:**

This study suggests that none of the provided options in a patient-led follow-up are unsuitable for patients who underwent curative surgery for primary CRC, based on coping style determined at baseline. Low-intensity surveillance after curative resection of CRC may, therefore, be suitable for a wide range of patients independent of coping styles.

**Supplementary Information:**

The online version contains supplementary material available at 10.1007/s00520-024-08758-y.

## Introduction

Colorectal cancer (CRC) is the third most common type of cancer in the Netherlands [1]. Surgery is a potentially curative treatment for approximately 90% of patients, although up to 30% develop postoperative disease recurrence [2]. For patients who present with metastases in the liver, lungs, or peritoneum, curative treatment options remain a possibility [3–6]. Consequently, patients are provided with follow-up care to facilitate early detection of asymptomatic and treatable recurrences.

In the Netherlands, CRC patients undergoing curative treatment are monitored for 5 years following surgery. National guidelines recommend clinical evaluation and carcinoembryonic antigen (CEA) measurements every 3 to 6 months during the first 2 years, followed by evaluation every 6 to 12 months during the last 3 years of follow-up. In addition, CT imaging of the chest and abdomen is recommended at 12 months of post-surgery [7].

Although extensively researched, there is no consensus on the ideal follow-up strategy [8–10]. Galjart et al. conducted a systematic review and meta-analysis demonstrating that more frequent follow-up, including tumour marker measurements and medical imaging, increased the rate of treatment with curative intent for recurrent disease. However, overall survival rates and cancer-specific survival rates are not affected by more intensive follow-up [8]. Follow-up care serves other purposes besides early detection of recurrence. It provides opportunities to assess patients’ psychosocial well-being, allows for diagnosis and treatment of (functional) complications such as neuropathy and symptoms of low anterior resection syndrome, and informs patients of their prognosis and disease status [11–17]. According to the results of a systematic literature review, less intensive follow-up after surgery for various types of cancer does not negatively affect the quality of life, emotional well-being, or patient satisfaction [18]. Moreover, frequent hospital visits can significantly affect patients' lives and can cause distress and fear of cancer recurrence [13, 19, 20]. Qaderi et al. performed a study exploring remote CRC follow-up, which resulted in high patient satisfaction and considerable cost savings [14].

Given the diversity of patients’ needs and preferences, more personalised follow-up strategies should be considered. It would be beneficial for physicians to be able to predict the optimal follow-up strategy for an individual patient. One potential factor for achieving this is through assessing patients’ coping styles.

Patients who are confronted with potentially threatening information are believed to adopt one of two coping styles, namely monitoring or blunting [21]. The monitoring coping style is characterised by cognitive confrontation and active information-seeking, whereas blunting is associated with cognitive avoidance and seeking distraction when facing a stressful situation [21, 22]. When faced with medical threats, patients with high monitoring tendencies exhibit more concern regarding their risks, actively seek out threatening health information, and worry more. Conversely, patients with low monitoring tendencies tend to avoid such reactions [23–27]. High blunting scores are associated with a decreased need for information [21, 26, 28]. After undergoing follow-up screening for an abnormal Pap smear, blunters were satisfied with the amount of information they received, but monitors would have preferred to know more [29]. Additionally, blunters reported fewer communication problems than monitors when receiving postoperative therapy for breast cancer [30]. Previous studies have shown that patients with a monitoring coping style prefer to be involved in decision-making regarding their medical treatments [23–26]. Furthermore, Miller et al. demonstrated that oncological patients achieve improved psychological and behavioural outcomes when the information they receive about their medical condition is tailored to their individual coping style.

This study aims to assess patient preferences concerning the frequency, blood sampling location, and communication of follow-up results among CRC patients. Secondly, it seeks to explore coping styles in medical scenarios of CRC patients and identify factors associated with these coping styles. Lastly, the study intends to examine the relationship between coping style and patients’ preferences for follow-up care. We hypothesise that patients with monitoring coping styles may show a preference for a more intensive frequency regarding blood sampling, blood sampling within a hospital setting, and direct communication with the treating physician.

## Methods

### Patient selection

The FUTURE-primary study is a prospective multicentre study, conducted among patients with curatively treated primary colorectal cancer, participating a patient-led, home-based follow-up programme [31]. In this patient-led home-based approach, participants have control over the frequency, blood sampling location, and communication of their postoperative surveillance. The study was approved by the Medical Ethics Committee of the Erasmus Medical Centre with the identification code NL77810.078.21 and is registered on clinicaltrials.gov under NCT05656326, registration date: 19/12/2022.

Patients were recruited from five Dutch hospitals: Erasmus Medical Centre (EMC), Leids Universitair Medisch Centrum (LUMC), IJsselland Hospital, Amphia Hospital, and Franciscus Hospital. Patients were eligible for inclusion if they had undergone surgery with curative intent for primary CRC and were scheduled for postoperative follow-up at one of the participating hospitals. Patients could be included up to six months postoperatively. Exclusion criteria were a severely complicated postoperative course requiring in-hospital follow-up for more than 6 months; enrolment in other studies or comorbidities requiring strict adherence to any specific follow-up with regular imaging of the abdomen and/or thorax; and inability to complete questionnaires due to illiteracy and/or insufficient proficiency in the Dutch language. Patients were asked to provide informed consent and complete the baseline questionnaires.

The standard follow-up frequency for patients within the study was every 6 months for the first 2 years, followed by yearly check-ups for the next 3 years (minimal frequency). In this study, patients were given the option of more frequent check-ups (maximal frequency), involving CEA assessments every 3 months for the first 2 years and every 6 months for the following 3 years.

The Dutch guideline provides a flexible interval for CEA measurements, leading to a variation in the standard of care among hospitals. In the Erasmus MC, LUMC, IJsselland Hospital, and Franciscus Hospital, the local standard of care involves maximal CEA measurements. In contrast, at the Amphia Hospital, the standard of care adheres to a minimal frequency.

In addition to these options, patients were asked to indicate their preference regarding the location of blood sampling. Patients had the option to choose self-performed lancet capillary sampling at home; venipuncture performed at their general practitioner’s office, going to a medical centre nearby, or visiting the hospital. Finally, patients were asked to indicate their preferred mode of communication with their physician for receiving results of their CEA blood sampling during follow-up. They were given the following options: physical consultation in the outpatient clinic (face-to-face), telephonic control or a silent control, where no contact would be made in case of non-aberrant results. Patients from all participating hospitals had access to their own electronic patient files at all times.

Sociodemographic and clinical characteristics were collected, including age, sex, educational level, (neo)-adjuvant therapies, and ASA-score, from both medical records and completed questionnaires. Individual patient information was stored in the web-based data management system Castor EDC, (Amsterdam, the Netherlands). The study data were collected by members of the research team directly at the study centre, specifically the first four authors. Data from the EMC and IJsselland Hospital were gathered by KV and MG. LW and MG collected data from the Amphia Hospital. Data from LUMC and Franciscus Hospital were collected by MvD.

### Coping style

To assess whether individuals adopt an information-seeking (monitoring) or information-avoiding (blunting) coping style, the Threatening Medical Situations Inventory (TMSI) questionnaire was included in the baseline questionnaire [22]. This is a tool designed to assess an individual’s anxiety and fear in medical situations. It is used to identify individuals who may experience high levels of anxiety and distress during medical procedures [22]. This validated questionnaire consists of four hypothetical medical threatening scenarios, each followed by six statements. Three of these statements relate to a monitoring coping style, while the other three relate to a blunting coping style. Each statement is scored on a five-point Likert scale (1 = not at all applicable; 5 = strongly applicable). Scores are obtained by summing up the items for both scales separately, resulting in two scores ranging from 12 to 60. In case of missing values, the individual’s average score for the relevant subscale (monitoring or blunting) was used if 50% or more of the items on the subscale were filled out. Firstly, the coping style subscales were dichotomised to high or low monitoring/blunting, using the mean score for each subscale as the cut-off value. Additionally, associations between the dominant coping style of an individual (i.e. either monitoring or blunting) and follow-up preferences were analysed.

### Statistics

Descriptive statistics were presented as medians and percentiles for continuous variables, and frequencies and percentages for categorical variables. Additionally, for coping style, the mean score and standard deviation were provided to define the cut-off value to dichotomise low and high coping styles. Internal consistency for the TMSI monitoring and blunting scales was assessed using Cronbach’s alpha, where scores above 0.7 are considered to be satisfactory and scores of 0.8 or greater are preferred [32]. Logistic regression analyses were used to compute associations between follow-up preferences and coping style, as well as other variables. Linear regression analyses were used to calculate the association between monitoring and blunting scores and participant characteristics. Variables with a significance level of *p* < 0.10 in the bivariate analysis were included in multivariate regression analysis. For all other analyses, a significant difference was indicated with a *p* < 0.05. Analyses were conducted using SPSS version 28 (SPSS, Chicago, IL, USA).

## Results

### Study population

From October 2021 to February 2024, a total of 200 patients were included in the FUTURE-primary study. Seven patients chose not to participate in the questionnaire, and an additional five patients were excluded due to inadequate responses on the TMSI questionnaire (i.e. less than 50% completed). A total of 188 were included for coping style analysis. Table 1 provides an overview of demographic and clinical characteristics, as well as follow-up preferences. Median age of the total cohort (*n* = 200) was 68 years (IQR 59–75) and 106 participants (53%) were male. The median monitoring score was 31.0 (out of 60) (IQR 23–39) and the median blunting score 34.0 (IQR 28–39).

**Table 1:** Baseline and clinical characteristics of study participants

**Characteristics**			
		No	Percentage
Age, years (Median, IQR)		68.0	59.0–74.8
Gender	Male	106	53.0
	Female	94	47.0
Educational level	Non-college graduated	130	65.0
	College graduated	64	32.0
	*Missing*	6	3.0
Coping styles scores (median, IQR)	Monitoring score	31.0	23–39
	Blunting score	34.0	28–39
Coping styles scores (mean, SD)	Monitoring score	31.2	10.3
	Blunting score	33.6	9.5
Dominant coping style	Monitoring	68	34.0
	Blunting	104	52.0
	Equal	16	8.0
	*Missing*	12	6.0
ASA-classification	ASA-1/ASA-2	129	64.5
	ASA-3/ASA-4	63	31.5
	*Missing*	8	4.0
Treating hospital	Centre A	66	33.3
	Centre B	12	6.0
	Centre C	12	6.0
	Centre D	102	51.0
	Centre E	8	4.0
Therapy	Neoadjuvant therapy*	27	13.5
	Adjuvant chemotherapy	21	10.5
	Only surgery	152	70.6
Control frequency	Maximal	143	71.5
	Minimal	57	28.5
Blood sampling location	Home	129	64.5
	Local diagnostics centre	44	22.0
	Hospital	27	13.5
Communication of follow-up results	Silent	53	26.5
	Telephone	138	69.0
	Physical	9	4.5

*Neoadjuvant chemotherapy and/or neoadjuvant radiotherapy

The internal consistencies, using Cronbach’s alpha, for the monitoring and blunting subscale were 0.886 and 0.884, respectively, making the TMSI a reliable questionnaire among CRC patients.

### Follow-up preferences

In total 143 (71.5%) participants chose for maximal control frequency. Blood sampling at home was chosen by 129 (64.5%) participants. In total 53 (26.5%) of the participants opted for silent controls. Surgeons propose a certain follow-up frequency in 118 patients identified through the electronic patient records, accounting for 59% of the total cohort. Only 11.5% of these patients opted to modify their initially proposed follow-up frequency after inclusion in the study.

### Coping styles

Among the patients whom completed the TMSI questionnaire sufficiently, 93 patients (49.5%) exhibit high monitoring styling. Conversely, 101 patients (53.7%) display high blunting scores. Bivariate linear regression analyses showed an association between a higher monitoring score and younger age, female gender, higher education levels, and lower ASA scores. These variables were included in the multivariate analysis, which showed significant associations between higher monitoring scores and a younger age (*B* =  − 0.172; *p* = 0.013), female gender (*B* = 4.241; *p* = 0.003), a higher educational level (*B* = 5.492; *p* < 0.001), and a lower ASA-score (*B* =  − 2.559; *p* = 0.032) (Table 3). For blunting scores, no significant association with any of the variables was found in linear regression analysis (Table 2).

**Table 2:** Results of bi- and multivariate linear regression analyses of factors influencing monitoring and blunting coping style

	Monitoring								Blunting							
	Bivariate		Multivariate						Bivariate		Multivariate					
	B	Sig	95% C.I		B	Sig	95% C.I. EXP(B)		B	Sig	95% C.I		B	Sig	95% C.I. EXP (B)	
			Lower	Upper			Lower	Upper			Lower	Upper			Lower	Upper
Age	− 0.251	< 0.001	− 0.387	− 0.116	− 0.172	**0.013**	− 0.308	− 0.036	− 0.044	0.500	− 0.174	0.085				
Gender (reference = male)	3.826	0.010	0.909	6.742	4.241	**0.003**	1.440	7.041	− 0.902	0.518	− 3.649	1.845				
Educational Level (reference = non-college educated)	5.772	< 0.001	2.726	8.819	5.492	** < 0.001**	2.514	8.471	0.712	0.632	− 2.212	3.637				
ASA-classification	− 3.922	0.001	− 6.282	− 1.562	− 2.559	**0.032**	− 4.893	− 0.225	− 0.246	0.830	− 2.007	2.499				
Neoadjuvant therapy	− 2.024	0.352	− 6.298	2.250					0.391	0.846	− 3.577	4.359				
Adjuvant chemotherapy	− 2.369	0.330	− 7.154	2.415					− 1.405	0.533	− 5.844	3.034				

The bold captured data is significant (*p* < 0.05)

### Frequency of CEA measurement

A higher age, standard of control frequency, an higher ASA-classification, and the preference for silent contact showed a bivariate association with the preference for a minimal intensive control frequency of CEA measurements (*p* = 0.003, *p* < 0.001, *p* = 0.036, and *p* = 0.084 respectively). These variables were included in a multivariate logistic regression analysis, only the standard follow-up frequency in the hospital was significantly associated with patients’ preferences in frequency of follow-up (OR = 5.35, 95% CI 2.26–10.02, *p* < 0.001) (Table 3).

**Table 3:** Results of bi- and multivariate logistic regression analyses of factors influencing surveillance preferences

	Control frequency (minimal)				Blood sampling location (home)				Control type (silent)			
	Bivariate	Multivariate			Bivariate	Multivariate			Bivariate	Multivariate		
	*p*-value	OR	95% CI	*p*-value	*p*-value	OR	95% CI	*p*-value	*p*-value	OR	95% CI	*p*-value
Age	**0.003**	1.02	0.99–1.06	0.222	**0.016**	0.96	0.93–0.99	**0.007**	**0.051**	0.97	0.94–1.00	**0.045**
Gender (reference = male)	0.382				0.319				0.117			
Educational level (reference = non-college educated)	0.611				0.574				0.223			
Standard of control frequency in treating hospital	** < 0.001**	5.35	2.62–10.92	** < 0.001**								
Monitoring score	0.544				0.118				0.370			
Blunting score	0.449				0.291				0.578			
ASA-classification	**0.036**	2.11	1.00–4.47	0.051	0.164				0.577			
Neoadjuvant therapy	0.750				0.267				0.942			
Adjuvant chemotherapy	0.994				0.485				0.417			
Control frequency, reference = minimal	Not included				0.366				**0.084**	1.66	0.83–3.35	0.153
Blood sampling location, reference = home	0.366				Not included				**0.054**	2.40	1.15–5.00	**0.020**
Communication of follow-up results, reference = silent	**0.084**	0.57	0.27–1.23	0.150	** < 0.001**	0.43	0.21–0.89	**0.022**	Not included			

The bold captured data is significant (*p* < 0.05)

### Blood sampling location

In the multivariate logistic regression, a younger age and the preference for silent contact were associated with the preference of home-based blood withdrawal (OR = 0.96, 95% CI 0.93–0.99, *p* = 0.007 for age and OR = 0.43, 95% CI 0.21–0.89, *p* = 0.022 for the preference silent contact).

### Type of contact with treating physician

A younger age, maximal frequency of follow-up, and blood withdrawal outside the home environment showed a bivariate association with the preference for a silent control (*p* = 0.051, *p* = 0.084, *p* = 0.054, respectively), and were included in a multivariate logistic regression analysis. A younger age and blood withdrawal outside the home environment were significantly associated with patients’ preferences of silent controls (OR = 0.97, 95 CI% 0.94–1.00, *p* = 0.045, OR = 2.40, 95% CI 1.15–5.00, *p* = 0.020).

## Discussion

To our knowledge, the present study is the first investigating the role of coping style in a patient-led follow-up trajectory in which patients actively participate in determining their follow-up care. Previous research has primarily focused on monitoring and blunting coping styles in relation to the need for information and involvement in decisions. High monitoring scores have been associated with a greater need for information and involvement in treatment [23–26, 33], whereas higher blunting scores are linked to a decreased need for information [21, 26, 28].

In line with previously performed research [22, 25, 34–37], associations were found between higher monitoring scores and younger age, female gender, and a higher level of education. These associations emphasise the need to consider individual differences in coping styles when designing patient-centred approaches to healthcare. For instance, Plamann et al. reported that patients with monitoring coping styles prefer more information [36]. However, our study did not reveal any direct links between monitoring and blunting coping styles and the preferences patients expressed for their follow-up care, which included preferences for a maximal intensive follow-up frequency. The preference for telephonic or physical contact also showed no association with having a higher monitoring score. This suggests that while monitoring and blunting coping styles may influence how individuals desire information in healthcare settings, they may not directly correlate with specific preferences for follow-up care modalities.

While coping styles have been valuable in understanding a patient’s psychological response to illness, this study underlines the complexity of tailoring follow-up care based solely on monitoring or blunting coping styles. The absence of significant associations with follow-up preferences and the fact that only 11.5% of patients opted to modify the initially proposed follow-up frequency suggests that other factors may play pivotal roles in shaping patients' choices. These factors could include health literacy, communication between patients and physicians, socioeconomic constraints, social support, and previous experiences with the healthcare system. Unfortunately, these factors were not measured in our study [38].

The standard of care within the treating hospital is the only factor with the ability to predict a patient’s choice for frequency of blood measurement. Although a proposed follow-up trajectory by the treating physician or nurse practitioner was described in only 59% of the medical records, we assume that a larger proportion of participants have already been influenced during the preoperative or hospitalisation periods.

The hypothesis that patients with monitoring coping styles prefer a more intensive follow-up scheme, did not hold in this cohort. Instead, this may be exclusively associated with a need for more information. This study implies that the different follow-up modalities can be offered to all patients in patient-led follow-up trajectories. The results of the FUTURE-primary will reveal if different follow-up strategies lead to any change in the quality of life. Within this study, no reason was found to withhold the option of less intensive follow-up from any specific group of patients. Less intensive home-based follow-up as standard care will result in fewer hospital appointments and fewer diagnostic tests, which could lead to a reduction in societal healthcare costs and hospital workload. Additionally, patients have to travel to the hospital less often, resulting in time and cost saving on individual patient levels as well. Fewer hospital appointments could also contribute to reducing the anxiety commonly associated with frequent medical appointments.

### Limitations

The limitation in this study lies in the predetermined standard of care for CEA measurements. Ideally, patients would be able to consider their decision without the influence of the physicians, unless requested. However, given the postoperative setting, patients are likely already aware of the hospital’s standard of care at the time of inclusion. If a patient-led follow-up is adopted, and standard care becomes uniform across hospitals, physician influence may diminish. Further investigation into patient-led follow-up post-implementation could validate our study outcomes without the confounding variable of varying standard care across hospitals. Additionally, another limitation involves the lower rates of patient inclusion observed in three of the participating hospitals. This was primarily due to the academic setting of two hospitals and later start of recruitment in one hospital. Subgroup analysis by treating centre was not useful due to the limited sample size from some hospitals. Therefore, the impact of the treating centre on follow-up preferences cannot be reliably assessed in this study. Despite these variations in inclusion rates, the diverse patient population included in the study provides valuable insights into coping styles among CRC patients, supporting the relevance and applicability of our findings within this specific patient group.

## Conclusion

In conclusion, no significant associations between CRC follow-up preferences and patients’ coping styles were found. This suggests that none of the provided options in a patient-led follow-up, i.e. frequency of follow-up, location of blood withdrawal, and method of contact, are unsuitable for patients who underwent curative surgery for primary CRC, based on coping style determined at baseline. While this suggests a broad applicability of patient-led follow-up strategies, the ultimate determination of the universal suitability of these options awaits the comprehensive results of the FUTURE-primary study. This ongoing research will provide valuable insights into tailoring follow-up strategies to individual patient needs, preferences, and quality of life.

### Supplementary Information

Below is the link to the electronic supplementary material.

Supplementary file1 (DOCX 30 KB)

## Data Availability

The datasets generated during and analysed during the current study are available from the corresponding author on reasonable request.
